# Generative AI in Youth Mental Health Apps: Rapid Review

**DOI:** 10.2196/90589

**Published:** 2026-08-19

**Authors:** Helene Høgsdal, Henriette Kyrrestad, Sabine Kaiser

**Affiliations:** 1 UiT The Arctic University of Norway Faculty of Health Sciences Regional Centre for Child and Youth Mental Health and Child Welfare - North Tromsø Norway

**Keywords:** mental health apps, adolescents, young people, generative artificial intelligence, GenAI, artificial intelligence, AI

## Abstract

**Background:**

Mental health apps are frequently used as platforms for delivering digital health interventions to young people. New technology such as generative AI enables a wider range of engaging and more personalized features that can be included in mental health apps. However, there is limited insight into the opportunities for integrating generative AI into such apps.

**Objective:**

This rapid review aimed to provide a systematic overview of the possibilities of integrating generative AI into mental health apps for young people. Furthermore, the review aimed to report potential benefits and disadvantages of the integration of generative AI into such apps.

**Methods:**

A systematic search was conducted in 4 databases (PsycInfo, Embase, MEDLINE, and CINAHL) in November 2025 to identify studies that evaluated mental health apps with integrated generative AI. Eligible studies included primary research published between 2022 and 2025, involving a sample of young people aged 11 to 24 years, and written in either English or a Scandinavian language.

**Results:**

A total of 5 articles were included in the review. The most common method of integrating generative AI into mental health apps for young people was through chatbots. Overall, young people rated the apps as having good usability and quality. Some studies also provided data on effectiveness, showing promising results for outcomes such as depression, anxiety, and distress. None of the studies systematically assessed harmful effects using standardized methods, nor did they report any adverse outcomes related to mental health.

**Conclusions:**

Young people are generally positive about apps that include generative AI. There is some evidence suggesting that such tools may contribute to a preventive or health-promoting effect on young people’s mental health. However, the existing research is limited and characterized by methodological constraints. The lack of reported adverse mental health outcomes might reflect a lack of investigation rather than evidence of no harm. Further research should explore the potential short- and long-term effects of integrating generative AI into mental health apps for young people, as well as systematically mapping possible adverse events.

## Introduction

### Background

Mental health apps are a frequently used platform for delivering digital health interventions to youth [1]. These interventions are designed to provide support for users’ mental health and well-being through an app-based platform [2]. Traditionally, mental health apps designed for youth have included features such as mindfulness exercises, meditation, stress management tools, and mood-tracking options [1,3]. Several of these apps provide users with practical advice for dealing with various challenges and can, in some cases, act as a “digital bridge” to traditional mental health services by supporting continuity and self-management [4,5]. In recent years, technological advancements have expanded the possibilities for mental health apps, enabling the integration of features that were previously difficult or impossible to implement, for instance, AI and generative AI (GenAI), such as large language models (LLMs; eg, the generative pretrained transformer [GPT] series and Claude). This rapid development is considered a promising step forward in delivering more effective digital mental health care [6]. To fully realize the potential of these advancements, a broader exploration of how such tools can be integrated into mental health apps specifically designed for young people is essential. This includes examining the opportunities they present, as well as the potential benefits and challenges associated with their implementation.

### AI and GenAI

AI can be explained as machines’ ability to simulate human intelligence [7]. It was traditionally used to classify and structure existing information [8]. AI has evolved from being able to recognize patterns and analyze existing data to using this knowledge to generate new content, such as text, images, and real-time answers. This form of AI, known as GenAI, refers to computational techniques capable of creating new and meaningful content, for example, texts, pictures, or audio, based on training data [8]. This enables the technology to write, explain, create, translate, brainstorm, and adapt to various tasks without being specifically trained for them. For example, LLMs, where models are trained using existing information and online sources, can use these data to generate contextually relevant responses [8].

GenAI’s ability to generate meaningful and contextually relevant content can, in some cases, resemble human creativity and support natural and engaging interactions with users [9]. Thus, it is not surprising that many people tend to use GenAI to investigate mental health problems or to receive support for various challenges [10,11]. In particular, adolescents, a population that is familiar with digital platforms [12], seem to embrace the potential and possibilities provided by GenAI. A study by Brandtzaeg et al [13] showed that GenAI has become a big part of many young people’s lives. Many use it for personal support, for example, related to education and entertainment, but also for mental health purposes, and emotional support. In a nationally representative study from the United States, McBain et al [14] examined the extent to which adolescents use GenAI for advice or support during emotional distress. The study found that 13% had used GenAI in such situations, with the highest prevalence among young people aged 18 to 21 years. Among those who used GenAI, a large majority reported that the advice was perceived as helpful [14]. Furthermore, in a study by Skjuve et al [15], responses from both ChatGPT and mental health care professionals were compared among 123 adolescents. Even though adolescents recognized that both sources can be helpful, they were more likely to recommend answers generated by GenAI because they were perceived as more precise, more structured, and friendlier.

### GenAI in Mental Health Care

The fact that many individuals use GenAI and that young people prefer answers from such tools [15] indicates a potential for such technology to be integrated into mental health–promoting and preventive interventions. GenAI is increasingly being used in mental health care [16] and has shown potential across several mental health–related contexts, including education, the prediction of mental health problems or changes in mental health, and conversations with users aimed at providing mental health information or support [17-20]. In particular, the ability of chatbots powered by LLMs to generate humanlike responses has been highlighted as promising to provide individuals with personalized and effective mental health support [16,19]. GenAI can also be used to create visuals of, for example, emotions or mental states to promote insight and facilitate reflection on the user’s mental health [21,22]. Overall, tools with integrated GenAI might be engaging and provide users with easily accessible and personalized support. Siddals et al [19] found that users interacting with mental health chatbots perceive those powered by GenAI as more engaging, flexible, and capable of providing higher-quality advice than rule-based AI chatbots, that is, AI that provides information or knowledge using predefined rules (if X, then Y) [23]. Additionally, compared to human support, users highlight benefits such as availability, less judgment, and increased creativity when engaging with GenAI chatbots for mental health purposes [19].

However, Nagata et al [24] state that, even though GenAI has benefits such as being engaging for youth and the ability to offer accessible support, it also poses risks such as creating misinformation and providing adolescents with support that lacks empathy and authentic connections. Moreover, the use of GenAI has been shown to be potentially harmful as it can provide overly validating responses that may reinforce negative behaviors or ideas in users [25]. There have been documented cases where the use of advanced technologies such as LLMs has been linked to severe incidents, including the onset of psychosis or hallucinatory experiences in certain individuals [26]. The documented risks, combined with the fact that adolescence is a particularly vulnerable developmental period [27], make it especially important that GenAI tools intended for this target group are monitored for potential harms and include safety mechanisms to prevent unwanted or potentially harmful outcomes for this population’s mental health.

### Study Aims

The aim of this study was to conduct a rapid review of recently published research literature exploring the integration of GenAI into mental health apps for young people. Furthermore, this review aimed to investigate whether the included studies reported any benefits or whether they systematically assessed negative effects on young people’s mental health or adverse events. More specifically, the following three research questions (RQs) were addressed:

1. How is GenAI integrated into mental health apps developed for young people?
2. What are the potential benefits of integrating GenAI into mental health apps for young people?
3. What potential adverse outcomes are reported in studies evaluating mental health apps with GenAI for young people?

## Methods

### Overview

This rapid review was conducted in line with PRISMA-RR (Preferred Reporting Items for Systematic Reviews and Meta-Analyses extension for rapid reviews) recommendations [28]. The RQs and inclusion and exclusion criteria were predefined and followed throughout the review process. This method differs from a full systematic review in that the search was time limited from 2022 to the present and to articles in only 4 languages. The search strategy and inclusion and exclusion criteria were developed and agreed upon by all authors prior to screening. The initial search and screening were conducted by the first author. The second author independently screened a random sample of 112 titles and abstracts (approximately 26% of the records), with no discrepancies identified. All included studies were reviewed by all authors, and final inclusion of the articles was agreed upon through discussion. Data extraction was conducted by the first author and subsequently reviewed by the last author. The second reviewer independently assessed the extracted data, and any discrepancies were discussed and resolved through consensus.

### Information Sources and Search Strategy

Searches were conducted in 4 electronic databases (PsycInfo [Ovid], Embase [Ovid], MEDLINE [Ovid], and CINAHL [EBSCOhost]) to identify primary studies on how mental health apps for young people have integrated GenAI. Search terms were adapted to each database and included a list of synonyms related to “mental health apps” or “applications,” “artificial intelligence” or “generative artificial intelligence,” and “mental health” (eg, “[artificial intelligence OR machine learning OR generative AI OR large language model*] AND [mental health OR depression OR anxiety OR psychological distress] AND [mobile app* OR mental health app* OR digital intervention*]”). The full search strings used in each database can be found in Tables S1 to S4 in Multimedia Appendix 1 [29-34].

### Eligibility Criteria

Included sources were primary studies that evaluated one or more specific apps with integrated GenAI among young people (11-24 years). The chosen age range is consistent with recent conceptual definitions of the adolescent period, where adolescence encompasses development until the mid-20s [35]. The publication period was set from 2022 to the present (November 2025), reflecting the relevance of GenAI [9]. The included languages were English, Swedish, Danish, and Norwegian. Exclusion criteria comprised nonprimary studies (eg, reviews or meta-analyses), studies that evaluated an app solely among individuals younger than 11 years or adults older than 24 years, or studies where GenAI was not integrated as part of an app. When the type of AI was unclear, the corresponding authors were contacted for clarification. Studies were excluded if GenAI use could not be confirmed. Additionally, studies published in languages other than those specified above were excluded.

### Quality Assessment

The quality assessment of the included articles was performed using the Mixed Methods Appraisal Tool (MMAT) version 2018 [29], which enables appraisal across multiple study designs. The quality appraisal was conducted by the first author and subsequently reviewed by the last author. Any discrepancies in scoring between the reviewers were resolved through discussion. All studies were appraised according to the criteria relevant to their respective study designs. In line with MMAT guidance [29], no overall score was calculated, but each criterion was assessed individually. Table S5 in Multimedia Appendix 1 [29-34] provides the detailed quality assessments.

## Results

### Characteristics of the Included Studies

**Figure 1:**
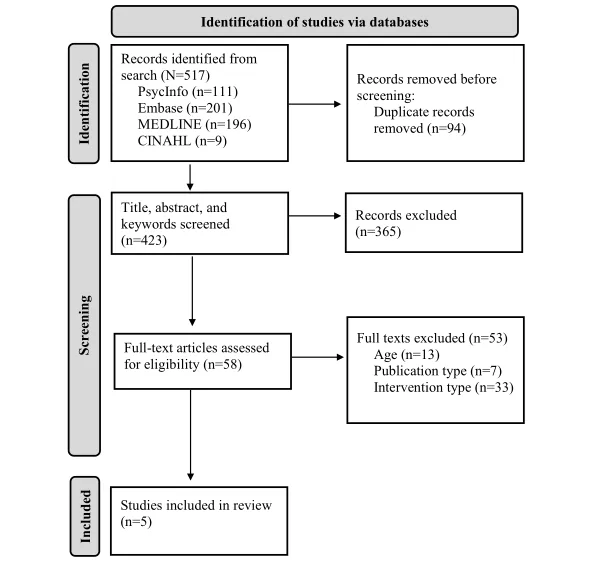
PRISMA (Preferred Reporting Items for Systematic Reviews and Meta-Analyses) diagram of study screening, review, and inclusion.

A total of 517 articles were identified in the initial search (Figure 1). Of these 517 articles, after removal of duplicates (n=94, 18.2%), 423 (81.8%) were screened by title and abstract. Of these 423 articles, 365 (86.3%) were excluded. A total of 58 articles were assessed for eligibility, and 53 (91.4%) were excluded for not meeting the inclusion criteria. For 3.4% (2/58) of the articles, the type of AI used was not specified, and the corresponding authors were contacted for clarification. Of these 2 articles, 1 was excluded due to lack of response as whether the intervention used GenAI could not be confirmed. In total, 5 studies were included in the review (Table 1). The included articles were conducted in the United States (3/5, 60%), China (1/5, 20%), and South Korea (1/5, 20%).

**Table 1:** Overview of the included studies.

Study	Country	Intervention	Study design^a^	Sample size, N^b^	Age range (y)	Age (y), mean (SD)	Outcome measures	Comparator	Follow-up duration	MMAT^c^ appraisal
Cohen et al [32], 2025	United States	Alongside app	Nonrandomized	116	10-18	14.02 (2.05)	Distress (YP-CORE^d^), depression (PHQ-2^e^), anxiety (GAD-2^f^), hopelessness (BHS-4^g^), loneliness (ULS-3^h^), and expectancies towards mental health treatment, and app use	None	1 and 3 mo	3/5
Emezue et al [34], 2024	United States	BrotherlyACT app	Mixed methods	15	15-24	21.00 (5.74)	Usability (SUS^i^) and user experiences (qualitative interviews)	None	None	4/5
Reyes-Portillo et al [33], 2025	United States	Wayhaven app	Nonrandomized	50	—^j^	22.12 (4.42)	Anxiety (GAD-7^k^), depression (PHQ-8^l^), hopelessness (BHS-4), agency (SHS^m^), self-efficacy (GSE^n^), well-being (SWEMWBS^o^), and engagement and satisfaction	None	1 session and 1 wk	2/5
Zhao et al [30], 2025	China	Douyin companion	RCT^p^	657	—	20.59 (2.00)	Depression (PHQ-9^q^), anxiety (GAD-7), and positive and negative affect (PANAS^r^)	Waitlist control group	2 and 4 wk	2/5
Lee et al [31], 2025	South Korea	Moa	RCT	75	—	20.42 (1.96, pooled)	Procrastination (PPS^s^ and IPS^t^), time management (TMBS^u^), academic self-regulation (ASRS^v^), stress (PSS-10^w^), engagement (in-app activity tracking), and usability (SUS)	App without chatbot	1 and 2 mo	4/5

^a^Study designs are classified according to the Mixed Methods Appraisal Tool [29].

^b^Sample sizes (N) reported in this table reflect the target population included in the analyses described in each study rather than the total baseline sample. Due to heterogeneity in study designs and reporting practices, N may vary across outcomes and time points.

^c^MMAT: Mixed Methods Appraisal Tool. MMAT scores are presented descriptively as the number of criteria met; no overall quality score was calculated.

^d^YP-CORE: Young Person’s Clinical Outcomes in Routine Evaluation.

^e^PHQ-2: Patient Health Questionnaire–2.

^f^GAD-2: Generalized Anxiety Disorder–2 scale.

^g^BHS-4: Beck Hopelessness Scale–4.

^h^ULS-3: University of California, Los Angeles, Loneliness Scale–3.

^i^SUS: System Usability Scale.

^j^not available.

^k^GAD-7: Generalized Anxiety Disorder–7 scale.

^l^PHQ-8: Patient Health Questionnaire–8.

^m^SHS: State Hope Scale.

^n^GSE: General Self-Efficacy Scale.

^o^SWEMWBS: Short Warwick-Edinburgh Mental Wellbeing Scale.

^p^RCT: randomized controlled trial.

^q^PHQ-9: Patient Health Questionnaire–9.

^r^PANAS: Positive and Negative Affect Schedule.

^s^PPS: Pure Procrastination Scale.

^t^IPS: Irrational Procrastination Scale.

^u^TMBS: Time Management Behavior Scale.

^v^ASRS: Academic Self-Regulation Scale.

^w^PSS-10: Perceived Stress Scale.

In line with the MMAT classification, 40% (2/5) of the articles were randomized controlled trials [30,31], 40% (2/5) were classified as quantitative nonrandomized [32,33], and 20% (1/5) used a mixed methods design [34]. In the articles included, anxiety and depression were the most frequently measured clinical outcomes [30,32,33], followed by stress and distress [31,32], hopelessness, and loneliness [32,33]. General mental well-being and psychological resources were also assessed, including well-being, affect, self-efficacy, and agency [30,33], as well as behavioral outcomes such as procrastination and self-regulation [31]. In addition, app-specific outcomes were assessed, particularly related to use and engagement, including frequency and duration of use as well as in-app activity [31-33]. Participants’ experiences with the app were also evaluated, including measures of usability [31,34], satisfaction or related user outcomes [32-34].

### GenAI in Mental Health Apps for Adolescents

Overall, the studies showed that GenAI in mental health apps was primarily implemented through conversational interfaces and named chatbots with specific and recognizable features [30-34] (Table 2). Among the studies that included LLMs within the apps, the underlying LLMs were based on the GPT series [34], Volcano Ark [30], and Claude [32]. Some studies also stated that they included hybrid or semigenerative systems combining rule-based and generative elements [31,32]. Although all the studies described built-in safety features such as crisis detection and referral mechanisms [30-34], none of the studies systematically assessed harmful effects as dedicated outcomes using standardized methods.

**Table 2:** Overview of the included interventions.

Intervention	Intervention objective	AI type	Interface	Safety features	Adverse mental health outcomes
Alongside	Provide personalized social-emotional learning and self-help wellness support to students while identifying those who need higher levels of support	Rule based+LLM^a^ (Claude)	Chatbot	Automated risk detection, crisis screening, referral to crisis support services, and human oversight	Not reported
BrotherlyACT	Reduce firearm injuries and homicides and improve access to precrisis and mental health resources for young Black male individuals in high-violence, low-resource settings	LLM (GPT-4.0)	Chatbot	Crisis resources and disclaimer stating that it is not a substitute for professional support	Not reported
Wayhaven	Deliver brief, evidence-based, personalized text-based support for college students’ mental health needs	Model not specified	Chatbot	Crisis resources, SOS button, and disclaimers stating that it is not for crisis use	Not reported
Douyin companion bot	Provide emotional companionship, encourage users to open up through empathetic dialogue, and alleviate negative emotions	LLM (Volcano Ark)	Chatbot	Crisis identification, referral to crisis support services, and human feedback training	Not reported
Moa	Foster users’ awareness of behavioral patterns, increase time management skills, and reduce procrastination	Hybrid or semigenerative (KoGPT2)	Chatbot	Limited topic, structured chatbot, and expert review	Not reported

^a^LLM: large language model.

The Alongside app aims to provide personalized, evidence-based mental health and social-emotional support for students [32]. In addition to rule-based AI, GenAI was integrated to provide users with validating statements and empathic responses. For instance, the app provides feedback with supportive and emphatic responses such as “I hear you Elsa. Not having friends can feel really tough” [32]. For the Alongside app, correspondence with the authors confirmed that the main chat function was based on Anthropic’s Claude architecture [32].

The BrotherlyACT app [34] aims to reduce the risk of firearm-related injuries and violence while improving access to preventive and mental health resources for young Black men in low-resource and high-risk settings. This app has integrated a chatbot named DEVON to support its users. The chatbot is built on GPT-4 to generate textual responses in dialogue with users based on their questions and inputs [34].

The Wayhaven app [33] includes a GenAI-based chatbot that aims to support college students’ mental wellness through personalized emotional support, tailored psychoeducational resources, and easy access to campus-specific support services. Within the chatbot, users can select an AI-based mental wellness coach. The conversations follow a structured session where the user identifies a problem and a goal before the chatbot offers evidence-based tools such as concrete breathing techniques or stress relief exercises for improving situations as well as helping create a concrete action plan [33].

Douyin’s companion bot is a chatbot integrated into the Douyin app based on the LLM Volcano Ark [30]. Users of the app can interact with the chatbot by clicking on “direct messages” on the home page of their Douyin app. The chatbot is designed to provide empathetic and accepting responses and functions primarily as an emotional companion rather than a clinical mental health service [30]. Furthermore, the model is explicitly geared toward present-focused interactions and avoids drawing attention to past experiences.

Moa is a semigenerative (ie, combined with predefined cognitive behavioral therapy–based scenarios to support behavior change) chatbot built into a to-do app to help students with procrastination and improve time management [31]. The input is analyzed through predefined cognitive behavioral therapy–based rules and classifications, whereas the responses of the chatbot are generated using an LLM based on KoGPT2 [31].

### Usability, Quality, and Engagement

A total of 3 studies reported data on how adolescents rated the apps’ usability or quality [31,33,34]. Among the studies that reported results on the System Usability Scale for the apps, the BrotherlyACT app received a score of 79, whereas the app containing the Moa chatbot received a score of 73, indicating that both apps had good usability [31,34]. In the study investigating young people’s use of the Wayhaven app, satisfaction with the app was measured immediately after using the app and after 1 week. The results showed that 90% found it easy to use, 74% were satisfied, 72% would use it again, 84% considered the app useful for the target group, and 53% found it engaging [33]. After 1 week, there was a general decrease in ratings, with 65% reporting satisfaction with the app, 59% stating that they would use the app again, 80% finding that it fit into everyday life, and 56% finding the app engaging [33].

### Potential Effects on Mental Health Outcomes

A total of 4 out of the 5 studies reported findings on potential effects of the apps on mental health outcomes [30-33].

#### Anxiety and Depression

The included articles examining the effects of the apps on anxiety and depression reported mixed findings. Among a general sample of adolescents, the Alongside intervention showed no significant effects on anxiety or depression at either the 1- or 3-month follow-up (*P*>.16 in all cases) [32]. Among the included studies specifically targeting participants with at least mild symptoms of anxiety or depression [30,33], participants using the Wayhaven app had a small reduction in anxiety and depression symptoms after 1 week (β=−2.15 and, β=−1.62 respectively) [33]. Participants in the intervention group using the Douyin companion chatbot did not exhibit a reduction in anxiety at 2 weeks; however, a small effect was observed at 4 weeks [30]. Reductions in depression scores were observed at both time points (2 and 4 weeks), although effect sizes were small to very small (η_p_^2^=0.016 and η_p_^2^=0.005, respectively) [30]. Furthermore, exploratory analyses of the Alongside app in a subsample of participants with higher levels of distress showed a statistically significant reduction in anxiety at 1 month [32].

#### Stress and Distress

Participants using the Alongside app experienced a small decrease in psychological distress at the 1-month follow-up; however, this effect was no longer observed at the 3-month follow-up [32]. The Moa chatbot did not affect stress levels at either the 1- or 2-month follow-up [31].

#### Other Mental Health and Behavioral Outcomes

Among the additional outcomes assessed in the included studies, the Alongside app demonstrated a moderate effect on hopelessness after 3 months [32]. In the study evaluating Wayhaven, participants showed a significant decrease in hopelessness after 1 week [33]. Improvements over time in agency, self-efficacy, and overall well-being were also observed among participants [33]. For the Moa chatbot, both groups showed some improvement over time; however, the treatment group demonstrated greater improvements in time management and larger reductions in procrastination [31].

### Disadvantages of GenAI in Mental Health Apps

None of the included studies reported adverse outcomes related to mental health when integrating GenAI into mental health apps. Some studies reported negative user experiences such as usability and navigation issues within the apps [34]. In addition, 39% of participants who conversed with Moa reported receiving illogical responses and described the chatbot as overly generic, lacking understanding, and providing limited support [31].

## Discussion

### Principal Findings

In this rapid review, 5 articles were included and reviewed to examine how GenAI is integrated into mental health apps developed for young people. The findings indicate that GenAI is most commonly integrated into mental health apps through chatbots. Although the current evidence base is still limited, the findings provide an initial overview of a rapidly emerging field and highlight important directions for future research.

Overall, the findings suggest that adolescents are satisfied with both the usability and quality of mental health apps with integrated GenAI. This finding is not surprising as young people often have high acceptability and hold positive attitudes toward GenAI and mental health apps in general [14,36]. GenAI can play an important role in communicating health information to young people by presenting it in a clear, easy-to-understand, and personalized way. At the same time, such solutions can provide easily accessible mental health support, which may be particularly relevant for young people who experience barriers related to waiting times or availability in traditional mental health services [37,38]. By providing personalized information and low-threshold emotional support, GenAI has the potential to make mental health services more accessible and scalable for young people [24].

Findings regarding the effectiveness of mental health apps with integrated GenAI were limited and varied. Some of the studies included in this review reported positive effects of the apps [30-33]. Despite small effect sizes, the findings may be relevant in a low-threshold and preventive context given the accessibility and scalability of such interventions [39,40]. The findings are in accordance with those of previous research indicating that GenAI can have positive effects on mental health outcomes. For example, a systematic review and meta-analysis of 14 randomized controlled trials showed that GenAI chatbots had a small to moderate effect in reducing mental health problems such as depression and anxiety in an adult population [41]. Similarly, a study investigating the Therabot app found that it was associated with moderate to large reductions in depressive symptoms, anxiety, and eating disorders among adults with existing clinical symptoms [42]. One study included in the present review also reported more favorable outcomes among LGBTQ participants using the Alongside app [32]. That some apps seem to be especially effective among minority groups is promising considering that these populations often experience more stigma and negative attitudes toward mental health services [43,44]. It is reasonable to believe that mental health apps and conversations with GenAI, where young people can experience anonymity and autonomy, can be perceived as less stigmatizing and, thus, also beneficial to this group [36].

Although none of the studies examining changes in mental health outcomes reported any deterioration in mental health, none systematically assessed or reported adverse events or severe adverse events, as recommended in previous research on monitoring harms in internet interventions [45]. This aligns with previous research showing that adverse events and potential risks related to mental health apps are rarely assessed or systematically reported in clinical trials despite evidence that such harms may occur in some users [46]. Because adolescence is a vulnerable developmental period during which many mental health problems emerge, the potential negative effects of GenAI on adolescents’ mental health are a key concern [24,47]. For example, adolescents might have problems distinguishing between AI-generated content and human communication, which can lead to misinformation and weaken genuine relationships [48]. Some studies also stress that GenAI can introduce health risks and worsen depressive symptoms or stress, as well as leading to sleep problems and reduced physical activity [24]. Furthermore, GenAI has been criticized for overvalidating users’ feelings or thoughts instead of following evidence-based strategies and for providing a false sense of empathy through humanlike responses without real understanding [49]. On the basis of the current evidence, it is not yet possible to draw firm conclusions regarding the effectiveness or potential adverse outcomes of GenAI-integrated mental health apps for young people. Overall, the findings of this review indicate that more research is needed on the potential disadvantages or harms, as well as the short- and long-term effects, of integrating GenAI into mental health apps for young people.

### Limitations

Although this rapid review followed the PRISMA-RR recommendations [28], several methodological limitations should be considered when interpreting the results. First, although partial double screening was conducted, not all records were independently screened, which may have introduced selection bias in the screening phase. Second, restricting the search to studies published from 2022 to the present may result in the exclusion of previously relevant studies that could have provided valuable insights. However, from 2022 onwards, advances in GenAI made these technologies increasingly accessible for public and user-driven applications [9], and therefore, the search should be sufficient to investigate how such technology can be integrated into mental health apps.

The studies included in this review were heterogeneous in their design, sample selection, and methods, with generally small sample sizes and often short follow-up periods. These factors limit the ability to draw conclusions and reduce the generalizability of the findings, particularly regarding the potential effects on mental health outcomes. Additionally, reporting of the underlying AI technology within the mental health apps was limited and inconsistent. Many studies referred to LLMs or AI-based conversational systems without specifying the exact model or architecture, limiting comparability and making it difficult to determine which technologies may underlie the reported effects. This underscores the need for more transparent reporting of underlying GenAI architectures in future research.

The use of a wide age range in the included articles (ie, from 11 to 24 years) may pose challenges for applicability. The age range was chosen in line with the definition of adolescence proposed by Sawyer et al [35]. However, this broad age span can encompass several distinct developmental stages, which may be associated with different risk profiles, patterns of technology use, and responses to interventions [50,51]. There is a need for more differentiated research that examines how GenAI in mental health apps impacts various age groups within adolescence. Despite this variation, the present review provides an exploratory overview of how GenAI is currently being integrated into mental health apps relevant to young people.

Finally, mental health apps with integrated GenAI may be available to young people without being evaluated. It is well known that only a limited number of mental health apps are systematically evaluated in empirical studies, and such evaluations are often resource intensive and time-consuming [52-54]. It is therefore plausible that apps with integrated GenAI used by young people were not captured in this review, either because they have not yet been evaluated in primary studies or because they are still under evaluation. This represents an important methodological limitation as it may lead to an underestimation of the extent of GenAI-based apps available to and used by young people.

### Conclusions

This rapid review provides an overview of how GenAI can be integrated into mental health apps designed for young people, as well as what advantages and disadvantages are reported by integrating this technology. The findings suggest that the most common application of GenAI in mental health apps is through chatbots and conversational interfaces. While evidence supporting the preventive or health-promoting effects of GenAI integrated into mental health apps for youth remains limited, some of the included studies suggest potential benefits, particularly for anxiety and depression. These effects appear to be most pronounced among groups with already elevated symptom burdens, highlighting the potential of such tools to be helpful for vulnerable groups. Moreover, the findings indicate that adolescents generally find mental health apps with GenAI both usable and engaging. This suggests that these apps are well designed and resonate with their target audiences, making them promising tools for supporting mental health. Further research should investigate the effect of such tools integrated into mental health apps, particularly in terms of their long-term impact on mental health outcomes. Furthermore, future studies should systematically explore and report potential risks or disadvantages of GenAI in mental health apps for young people.

## Data Availability

Data sharing is not applicable to this article as no datasets were generated or analyzed during this study.

## Appendix

Multimedia Appendix 1 Search strategy and completed MMAT quality assessments.

Multimedia Appendix 2 PRISMA checklist.

## Abbreviations

GenAI: generative artificial intelligence
GPT: generative pretrained transformer
LLM: large language model
MMAT: Mixed Methods Appraisal Tool
PRISMA: Preferred Reporting Items for Systematic Reviews and Meta-Analyses
PRISMA-RR: Preferred Reporting Items for Systematic Reviews and Meta-Analyses extension for rapid reviews
RQ: research question
