# Supplementary material for: Generative AI in Youth Mental Health Apps: Rapid Review
Source: JMIR Ment Health. 2026 Aug 19;13:e90589. doi: 10.2196/90589 (PMC13539175; doi:10.2196/90589)
Supplement: Supplementary file 2 [file mental_v13i1e90589_app1.docx]

**Multimedia Appendix 1**

**Table S1**

Search strings used in PsycInfo

| 1 | Artificial Intelligence/ | 16,008 |
| --- | --- | --- |
| 2 | Machine Learning/ | 15,782 |
| 3 | Generative Artificial Intelligence/ | 619 |
| 4 | (AI or artificial intelligence).ti,ab,id. | 20,131 |
| 5 | Machine learning.ti,ab,id. | 17,784 |
| 6 | (GenAI or generative AI or generative artificial intelligence).ti,ab,id. | 853 |
| 7 | (large language model* or LLM*).ti,ab,id. | 1,031 |
| 8 | or/1-7 | 45,302 |
| 9 | Mental Health/ | 108,743 |
| 10 | Mental Disorders/ | 104,921 |
| 11 | Well Being/ | 73,801 |
| 12 | Life Satisfaction/ | 14,918 |
| 13 | Stress/ | 85,060 |
| 14 | Subjective Well Being/ | 1,593 |
| 15 | Distress/ | 35,129 |
| 16 | Emotional Well Being/ | 607 |
| 17 | "Quality of Life"/ | 53,769 |
| 18 | (mental health or mental disorder* or mental illness*).ti,ab,id. | 373,151 |
| 19 | (psychological distress or psychological well-being or wellbeing or well-being).ti,ab,id. | 182,380 |
| 20 | (anxiety or anxiousness or depression or depressive).ti,ab,id. | 528,721 |
| 21 | (stress or distress or psychological stress).ti,ab,id. | 364,407 |
| 22 | (quality of life or QoL).ti,ab,id. | 101,588 |
| 23 | or/9-22 | 1,215,998 |
| 24 | Mobile Applications/ | 3,772 |
| 25 | Mobile Devices/ | 3,337 |
| 26 | Digital Interventions/ | 3,323 |
| 27 | Mobile Health Applications/ | 626 |
| 28 | Smartphones/ | 4,237 |
| 29 | (mobile app* or smartphone app* or phone app*).ti,ab,id. | 6,543 |
| 30 | (application or applications or app or apps or app-based).ti,ab,id. | 236,926 |
| 31 | (mobile health app* or mobile health application* or m-health or mhealth).ti,ab,id. | 3,343 |
| 32 | (mental health app* or MHapp* or psychology app* or health app*).ti,ab,id. | 6,051 |
| 33 | (digital mental health or digital intervention* or digital health intervention*).ti,ab,id. | 1,917 |
| 34 | or/24-33 | 249,582 |
| 35 | 8 and 23 and 34 | 1,058 |
| 36 | limit 35 to (("200 adolescence (age 13 to 17 yrs)" or "320 young adulthood (age 18 to 29 yrs)") and (danish or english or norwegian or swedish) and yr="2022 - 2025") | 111 |

**Table S2**

Search strings used in MEDLINE

| 1 | Artificial Intelligence/ | 60,149 |
| --- | --- | --- |
| 2 | Machine Learning/ | 61,413 |
| 3 | Generative Artificial Intelligence/ | 1,185 |
| 4 | (AI or artificial intelligence).ti,ab,kw. | 137,526 |
| 5 | Machine learning.ti,ab,kw. | 168,733 |
| 6 | (GenAI or generative AI or generative artificial intelligence).ti,ab,kw. | 3,256 |
| 7 | (large language model* or LLM*).ti,ab,kw. | 10,693 |
| 8 | or/1-7 | 309,226 |
| 9 | Mental Health/ | 75,557 |
| 10 | Mental Disorders/ | 189,363 |
| 11 | "Quality of Life"/ | 313,507 |
| 12 | (mental health or mental disorder* or mental illness*).ti,ab,kw. | 362,811 |
| 13 | (psychological distress or psychological well-being or emotional well-being or wellbeing or well-being).ti,ab,kw. | 223,022 |
| 14 | (anxiety or anxiousness or depression or depressive).ti,ab,kw. | 715,203 |
| 15 | (stress or distress or psychological stress).ti,ab,kw. | 1,299,526 |
| 16 | (quality of life or life satisfaction or QoL).ti,ab,kw. | 488,894 |
| 17 | or/9-16 | 2,726,594 |
| 18 | Mobile Applications/ | 15,992 |
| 19 | Smartphone/ | 12,353 |
| 20 | (mobile app* or smartphone app* or phone app*).ti,ab,kw. | 23,661 |
| 21 | (application or applications or app or apps or app-based).ti,ab,kw. | 2,015,043 |
| 22 | (mobile health app* or mobile health application* or m-health or mhealth).ti,ab,kw. | 14,871 |
| 23 | (mental health app* or MHapp* or psychology app* or health app*).ti,ab,kw. | 15,401 |
| 24 | (digital mental health or digital intervention* or digital health intervention*).ti,ab,kw. | 5,981 |
| 25 | or/18-24 | 2,041,180 |
| 26 | 8 and 17 and 25 | 4,181 |
| 27 | limit 26 to (yr="2022 -Current" and ("adolescent (13 to 18 years)" or "young adult (19 to 24 years)") and (danish or english or norwegian or swedish)) | 196 |

**Table S3**

Search strings used in Embase

| 1 | artificial intelligence/ | 126,977 |
| --- | --- | --- |
| 2 | machine learning/ | 186,400 |
| 3 | generative artificial intelligence/ | 4,222 |
| 4 | (AI or artificial intelligence).ti,ab,kw. | 176,326 |
| 5 | Machine learning.ti,ab,kw. | 195,148 |
| 6 | (GenAI or generative AI or generative artificial intelligence).ti,ab,kw. | 4,726 |
| 7 | (large language model* or LLM*).ti,ab,kw. | 12,255 |
| 8 | or/1-7 | 409,383 |
| 9 | mental health/ | 288,229 |
| 10 | mental disease/ | 377,558 |
| 11 | wellbeing/ | 101,635 |
| 12 | life satisfaction/ | 15,337 |
| 13 | emotional well-being/ | 7,116 |
| 14 | "quality of life"/ | 817,655 |
| 15 | (mental health or mental disorder* or mental illness*).ti,ab,kw. | 462,883 |
| 16 | (psychological distress or psychological well-being or wellbeing or well-being).ti,ab,kw. | 284,028 |
| 17 | (anxiety or anxiousness or depression or depressive).ti,ab,kw. | 1,038,027 |
| 18 | (stress or distress or psychological stress).ti,ab,kw. | 1,679,928 |
| 19 | (quality of life or QoL).ti,ab,kw. | 801,248 |
| 20 | or/9-19 | 3,987,606 |
| 21 | mobile application/ | 31,667 |
| 22 | mobile health application/ | 7,285 |
| 23 | smartphone/ | 42,016 |
| 24 | (mobile app* or smartphone app* or phone app*).ti,ab,kw. | 34,178 |
| 25 | (application or applications or app or apps or app-based).ti,ab,kw. | 2,355,764 |
| 26 | (mobile health app* or mobile health application* or m-health or mhealth).ti,ab,kw. | 15,864 |
| 27 | (mental health app* or MHapp* or psychology app* or health app*).ti,ab,kw. | 18,311 |
| 28 | (digital mental health or digital intervention* or digital health intervention*).ti,ab,kw. | 6,369 |
| 29 | or/21-28 | 2,403,671 |
| 30 | 8 and 20 and 29 | 6,493 |
| 31 | limit 30 to (yr="2022 -Current" and adolescent <13 to 17 years>) | 201 |

**Table S4**

Search strings used in CINAHL

| 1 | MH "Mobile Applications" OR MH "Smartphone" OR ((mobile app* or smartphone app* or phone app*):TI,AB) OR ((application or applications or app or apps or app-based):TI,AB) OR ((mobile health app* or mobile health application* or m-health or mhealth):TI,AB) OR ((mental health app* or MHapp* or psychology app* or health app*):TI,AB) OR ((digital mental health or digital intervention* or digital health intervention*):TI,AB) |
| --- | --- |
| 2 | MH "Mental Health" OR MH "Mental Disorders" OR well-b OR MH "Psychological Well-Being" OR MH "Quality of Life" OR ((mental health or mental disorder* or mental illness*):TI,AB) OR ((psychological distress or psychological well-being or wellbeing or well-being):TI,AB) OR ((anxiety or anxiousness or depression or depressive):TI,AB) OR ((stress or distress or psychological stress):TI,AB) OR ((quality of life or QoL):TI,AB) |
| 3 | MH "Artificial Intelligence" OR MH "Machine Learning" OR MH "Artificial Intelligence, Generative" OR ((AI OR "artificial intelligence"):TI,AB) OR Machine learning:TI,AB OR ((GenAI or generative AI or generative artificial intelligence):TI,AB) OR ((large language model* or LLM*):TI,AB) |
| 4 | (MH "Mobile Applications" OR MH "Smartphone" OR ((mobile app* or smartphone app* or phone app*):TI,AB) OR ((application or applications or app or apps or app-based):TI,AB) OR ((mobile health app* or mobile health application* or m-health or mhealth):TI,AB) OR ((mental health app* or MHapp* or psychology app* or health app*):TI,AB) OR ((digital mental health or digital intervention* or digital health intervention*):TI,AB)) AND (MH "Mental Health" OR MH "Mental Disorders" OR well-b OR MH "Psychological Well-Being" OR MH "Quality of Life" OR ((mental health or mental disorder* or mental illness*):TI,AB) OR ((psychological distress or psychological well-being or wellbeing or well-being):TI,AB) OR ((anxiety or anxiousness or depression or depressive):TI,AB) OR ((stress or distress or psychological stress):TI,AB) OR ((quality of life or QoL):TI,AB)) AND (MH "Artificial Intelligence" OR MH "Machine Learning" OR MH "Artificial Intelligence, Generative" OR ((AI OR "artificial intelligence"):TI,AB) OR Machine learning:TI,AB OR ((GenAI or generative AI or generative artificial intelligence):TI,AB) OR ((large language model* or LLM*):TI,AB))’ |

**Table S5**

Quality appraisal of included studies

|  | **First author (year)** | | | | |
| --- | --- | --- | --- | --- | --- |
|  | Cohen et al [32], (2025) | Emezue et al [34], (2024) | Reyes-Portillo et al [33], (2025) | Zhao et al [30], 2025 | Lee et al [31], 2025 |
| **Quantitative non-randomized** |  |  |  |  |  |
| Representative sample | No |  | No |  |  |
| Appropriate measurements | Yes |  | Yes |  |  |
| Complete outcome data | No |  | n/a |  |  |
| Confounders accounted for | Yes |  | No |  |  |
| Intervention delivered as intended | Yes |  | Yes |  |  |
| **Mixed methods** |  |  |  |  |  |
| Rationale for MM design |  | Yes |  |  |  |
| Effective integration of components |  | Yes |  |  |  |
| Integration outputs interpreted |  | Yes |  |  |  |
| Divergences adequately addressed |  | No |  |  |  |
| Adherence to methodological quality |  | Yes |  |  |  |
| **Quantitative randomized controlled trials** |  |  |  |  |  |
| Appropriate randomization |  |  |  | No | Yes |
| Baseline comparability |  |  |  | Yes | Yes |
| Complete outcome data |  |  |  | No | No |
| Blinded outcome assessment |  |  |  | No | Yes |
| Intervention adherence |  |  |  | Yes | Yes |
| **Total MMAT Criteria Met** | 3/5 | 4/5 | 2/5 | 2/5 | 4/5 |

Note. Abbreviated MMAT criteria are presented; full item wording is available in the Mixed Methods Appraisal Tool (MMAT) version 2018 [29].

**References**

29.	Hong QN, Fàbregues S, Bartlett G, Boardman F, Cargo M, Dagenais P, et al. The Mixed Methods Appraisal Tool (MMAT) version 2018 for information professionals and researchers. Education for Information. 2018;34(4):285–91. <https://doi.org/10.3233/efi-180221>

30.	Zhao Y, Qian W, Chen Y, Wu D, Luo Y, Gao C, et al. Effect of an AI agent trained on a large language model (LLM) as an intervention for depression and anxiety symptoms in young adults: A 28-day randomized controlled trial. Applied Psychology: Health and Well-Being. 2025;17(5):e70067. <https://doi.org/10.1111/aphw.70067> PMID:40910958.

31.	Lee S, Jeong J, Kim M, Lee S, Kim SP, Jung D. Development of a Mobile Intervention for Procrastination Augmented With a Semigenerative Chatbot for University Students: Pilot Randomized Controlled Trial. JMIR MHealth and UHealth. 2025;13:e53133. <https://doi.org/10.2196/53133> PMID:40208664.

32.	Cohen K, Rapoport A, Friis E, Hill S, Feldman S, Schleider J. The Alongside Digital Wellness Program for Youth: Longitudinal Pre-Post Outcomes Study. JMIR Formative Research. 2025;9:e73180. <https://doi.org/10.2196/73180> PMID:41061253.

33.	Reyes-Portillo JA, So A, McAlister K, Nicodemus C, Golden A, Jacobson C, et al. Generative AI-Powered Mental Wellness Chatbot for College Student Mental Wellness: Open Trial. JMIR Formative Research. 2025;9:e71923. <https://doi.org/10.2196/71923>

PMID:40726405.

34.	Emezue C, Dan-Irabor D, Froilan A, Dunlap A, Zamora P, Negron S, et al. Evaluating an App-Based Intervention for Preventing Firearm Violence and Substance Use in Young Black Boys and Men: Usability Evaluation Study. JMIR Formative Research. 2024;8:e60918. <https://doi.org/10.2196/60918> PMID:39589765.
